# Long-Read Sequencing is Required for Precision Diagnosis of Incontinentia Pigmenti

**DOI:** 10.21203/rs.3.rs-5811417/v1

**Published:** 2025-01-30

**Authors:** Monica H. Wojcik, Robin D. Clark, Abdallah F. Elias, Casie A. Genetti, Jill A. Madden, Dana Simpson, Linda Golkar, Miranda PG Zalusky, Angela L. Miller, Araceli Rodriguez, Joy Goffena, Camille A. Dash, Nikhita Damaraju, Sophia B. Gibson, Sophia HR Storz, Zach Anderson, Jonas A. Gustafson, Isabelle Thiffault, Emily G. Farrow, Tomi Pastinen, Jasmine Lin, Jennifer Huang, Alan H. Beggs, Pankaj B. Agrawal, Genomics Research to Elucidate the Genetics of Rare Diseases (GREGoR) Consortium, David T. Miller, Danny E. Miller

**Affiliations:** 1 Boston Children’s Hospital and Harvard Medical School, Boston, MA; 2 Division of Clinical Genetics, Loma Linda University Children’s Hospital and Loma Linda University School of Medicine, Loma Linda, CA; 3 Department of Medical Genetics, Shodair Children’s Hospital, Helena, MT 59601,; 4 Division of Biological Sciences, University of Montana, Missoula, MT 59812, USA.; 5 Division of Medical Genetics, Department of Pediatrics, University of Utah, Salt Lake City, UT 84112; 6 Randall Children’s Hospital, Portland, OR; 7 Division of Genetic Medicine, Department of Pediatrics, University of Washington and Seattle Children’s Hospital, Seattle, WA; 8 Department of Genome Sciences, University of Washington, Seattle, WA; 9 Molecular and Cellular Biology Program, University of Washington, Seattle, WA; 10 Children’s Mercy Hospital, Kansas City, MO; 11 Department of Laboratory Medicine and Pathology, University of Washington, Seattle, WA; 12 Brotman Baty Institute for Precision Medicine, University of Washington, Seattle, WA

## Abstract

Incontinentia pigmenti (IP) is caused by loss-of-function variants in *IKBKG*, with molecular genetic diagnosis complicated by a pseudogene. We describe seven individuals from three families with IP but negative clinical testing in whom long-read sequencing identified causal variants. Concurrent methylation analysis explained disease severity in one individual who died from neurologic complications, identified a mosaic variant in an individual with an atypical presentation, and confirmed skewed X-chromosome inactivation in an XXY individual.

## MAIN TEXT

Incontinentia pigmenti (IP, MIM 308310) is an X-linked disorder caused by loss-of-function variants in *IKBKG* that is typically male (XY) lethal, with phenotypic heterogeneity in females (XX) caused by variable X-chromosome inactivation.^1^ Genetic diagnosis of IP is complicated by the presence of a pseudogene, *IKBKGP1*, making it difficult for currently available clinical tests to accurately sequence the region and identify disease-causing variants.^2,3^ Long-read sequencing (LRS) is an emerging technique that is better able to evaluate these complex genomic regions due to the length of the reads generated compared to short-read approaches.^4^ Using LRS, we evaluated seven individuals from three families with a clinical diagnosis of IP but negative clinical testing, including an individual with Kleinfelter syndrome. We identified variants missed by standard testing, provided insight into disease severity in one individual, and demonstrated how LRS can be used for comprehensive clinical evaluation of complex genomic regions.

**Figure 1.**
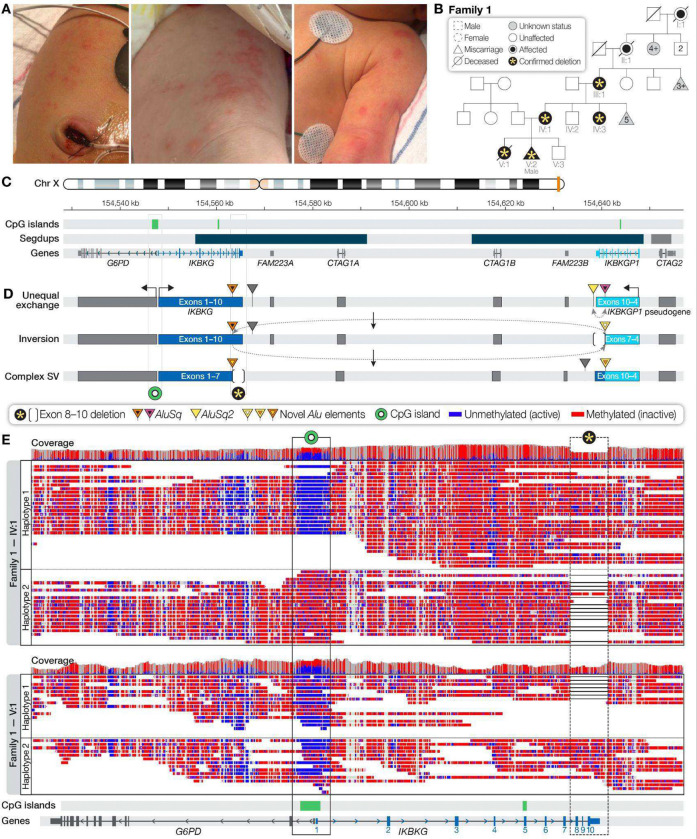
A deletion of *IKBKG* exons 8–10 likely resulted from an inversion. (**A**) The neonate displayed erythematous papules and vesicles in a Blaschkoid pattern on the abdomen, legs, and arms, consistent with the early findings of IP. (**B**) Pedigree consistent with X-linked inheritance of a XY male-lethal disorder. The neonate’s mother, maternal aunt, and grandmother had received a clinical diagnosis of IP, which was also suspected in the maternal great-grandmother and great-great-grandmother given their clinical history, recurrent miscarriages, and predominantly XX female offspring. (**C**) Structure of the *IKBKG* locus showing the position of *IKBKG*, two large segmental duplications, and the pseudogene *IKBKGP1*, which contains nearly identical copies of *IKBKG* exons 3–10. (**D**) The deletion identified in the family likely arose via a two-step mechanism. First, an unequal exchange event between *Alu* transposable elements (triangles) at *IKBKGP1* removed exons 8–10 of the pseudogene. Second, an exchange event between the *Alu* in intron 7 of *IKBKG* and the remaining *Alu* in *IKBKGP1* resulted in an inversion of the locus, which moved exons 8–10 of *IKBKG* to *IKBKGP1*. (**E**) Phased LRS data shows skewed X-inactivation over a CpG island within *IKBKG* (box) in the mildly affected mother (IV:1), but random X-inactivation in the more severely affected proband (V:1). Methylation status at CpG islands directs the expression of the gene at that site; methylated (inactive) CpG sites are shown in red and unmethylated (active) CpG sites in blue. The familial exon 8–10 deletion is also indicated (dashed box). Numbers indicate *IKBKG* exons.

In Family 1, a female neonate (V:1) who presented with seizures, strokes, and a Blaschkolinear rash characteristic of IP (Fig 1A) died in the setting of severe neurological complications at 13 days of age (Supplement). Although the neonate’s mother (IV:1), maternal aunt (IV:3), and maternal grandmother (III:1) had been clinically diagnosed with IP (Fig 1B), prior clinical genetic testing, including both targeted evaluation of *IKBKG* and exome sequencing, was negative. We performed research LRS using blood-derived DNA from the proband and her mother, aunt, and grandmother and detected a complex structural variant in *IKBKG* composed of a ~2,350-base-pair deletion that included exons 8–10 flanked by an ~80-kbp inversion (Fig 1C,D). Reads spanning this region indicated that the causal variant likely originated in a distant relative after recombination between *Alu* transposable elements in *IKBKG* and *IKBKGP1*. Custom long-range PCR clinically confirmed the variant in the neonate’s mother and grandmother (Supplement) and in a subsequent male miscarriage (V:2) (Fig 1B). Because LRS also captures methylation status, which can be used to evaluate X-inactivation patterns, we determined that the mildly affected female family members had complete inactivation of the haplotype with the deletion while the affected neonate had variable inactivation (Fig 1E, Supplement). The family has had a healthy male infant that was conceived spontaneously and tested negative for the *IKBKG* variant postnatally and is now using this information for preimplantation genetic testing.

**Figure 2.**
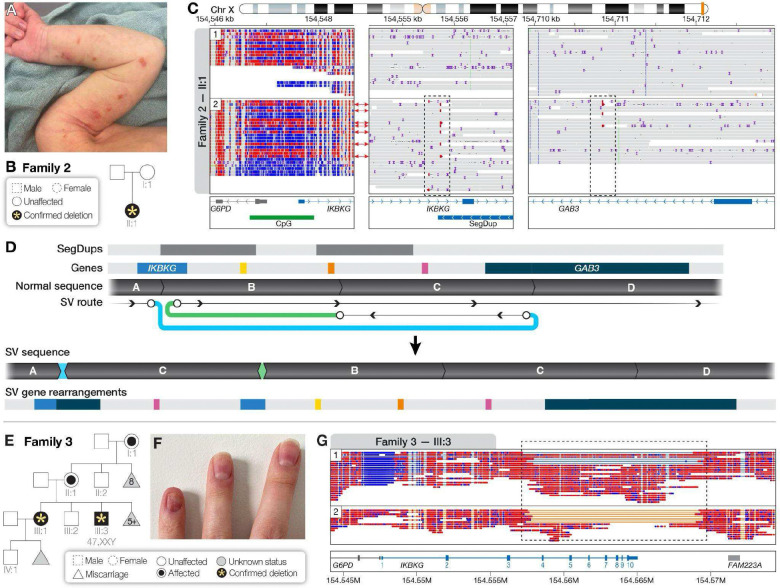
Disease-causing variation missed by prior clinical testing in two families with IP. **(A–D)** Family 2. **(A)** The proband (II:1) displayed a rash consistent with IP at birth. **(B)** The proband was the only affected individual in the family, thus a *de novo* variant was suspected. **(C)** Phased IGV view of LRS data with relevant gene regions shown. An atypical inversion bisects *IKBKG* and *GAB3* (dashed boxes) on the paternal haplotype (2). Arrows indicate reads that span from the promoter region of *IKBKG* to the inversion breakpoint within *IKBKG* and show that these reads are methylated. The inversion breakpoint within an intron of *GAB3* creates a small duplication, leaving one intact copy of *GAB3* on the affected haplotype. **(D)** Subway plot demonstrates the structure of the complex SV, which includes the inversion and duplication. **(E–G)** Family 3. **(E)** Multiple females from Family 3 with clinical diagnoses of IP also had miscarriages. The proband (III:1) had a mildly affected male sibling who was found to be 47,XXY. **(F)** A photo of the proband’s left hand shows abnormal nail beds. **(G)** Analysis of LRS data from individual III:3 (shown) as well as III:1 (not shown) revealed an 11.7-kbp deletion and skewed X-inactivation. Some reads in individual III:3 carrying the deletion are assigned to haplotype 1 as a result of poor phasing of shorter fragments generated using DNA isolated from saliva.

The proband (II:1) from Family 2 was a 2-year-old female diagnosed with IP in infancy based on physical findings of linear papules with hyperpigmentation and necrotic keratinocytes by punch biopsy at 14 days of age (Fig 2A). Because the proband was the only affected member of the family, a *de novo* variant in *IKBKG* was suspected but targeted clinical testing was negative. We performed research LRS of blood-derived DNA from the proband and her unaffected mother (I:1) and identified an atypical inversion bisecting *IKBKG* in the proband only (Fig 2B). Evaluation of phased reads from the proband revealed that the 27 reads spanning the inversion breakpoint and assigned to haplotype 1 were from the maternally derived X chromosome and showed no evidence of an inversion. In contrast, 6 of 21 reads assigned to haplotype 2 and 7 of 7 unphased reads showed evidence of an inversion, suggesting that 13 of 55 reads (24%) were likely from a chromosome carrying an inversion (Fig 2C). Reads that were split from those mapping to the left side of the inversion breakpoint within *IKBKG* also mapped to an intron of *GAB3*, suggesting an approximately 150-kb inversion event that duplicated the proximal part of *GAB3* (Fig 2D). Evaluation of methylation across both haplotypes revealed a mixed methylation pattern at the CpG island shared by *G6PD* and *IKBKG* for both haplotypes, but all 7 reads from haplotype 2 that were from the chromosome with the inversion were methylated, suggesting selection against chromosomes carrying this inversion during development (Fig 2C). Analysis of local methylation patterns in both the proband and mother revealed no skewed X-inactivation in either individual (Fig S5). Identification of an atypical mosaic inversion on the paternally derived chromosome allowed for more accurate counseling regarding recurrence risk for this family. The family declined clinical confirmation of the inversion for cost reasons.

Family 3 was identified during preconception evaluation of a 25-year-old female with personal and maternal family histories of IP. IP had been clinically diagnosed over three generations, affecting the proband (III:1), her mother (II:1), maternal grandmother (I:1), and a brother (III:3) who also had Klinefelter syndrome (47,XXY) (Fig 2E). The proband showed classic IP skin findings of blistering at birth progressing to hyperkeratotic lesions and swirling hyperpigmented patterns along Blaschko’s lines. In adulthood, she had sparse eyelashes/eyebrows, nail ridges (Fig 2F), hypodontia, and linear hypopigmented atrophic patches, but normal growth and development without neurologic or significant ophthalmic issues. Clinical genetic testing was negative, and X-chromosome inactivation was extremely skewed. Her younger brother (III:3) also presented with a characteristic IP rash from birth. A skin biopsy confirmed IP, and karyotype showed 47,XXY. Both the mother and maternal grandmother exhibited typical IP skin changes and hair/nail abnormalities, with multiple miscarriages reported. Targeted LRS of the proband and affected brother revealed an ~11.7-kbp deletion that included exons 4–10 of *IKBKG* (Fig 2G), and both individuals had skewed X-inactivation (Fig S5B). The deletion breakpoints overlapped two LTRs annotated as ERV1 elements (MER67B) by RepeatMasker that were also annotated as self-chain pairs (Fig S6), suggesting the deletion arose by unequal exchange between these homologous sequences.^5^

In summary, we describe three families with X-linked IP, all of whom were clinically diagnosed with this condition but lacked molecular confirmation due to the limitations of commonly available clinical short-read technologies, such as exome, single gene, or panel sequencing. Despite high suspicion for IP based on clinical features, a molecular diagnosis was desired for reproductive planning and counseling due to the high recurrence risk for affected mothers. Using LRS, we identified the causal variants for all three families, including a likely mechanism underlying the formation of the variants in Families 1 and 3 and an explanation for why the proband in Family 1 was more severely affected than other females in the family. This information enabled Families 1 and 3 to pursue *in vitro* fertilization with preimplantation genetic testing to avoid transmission in future pregnancies, while Family 2 received accurate counseling regarding recurrence risk for their future offspring. These cases demonstrate the utility of LRS, as a single test, to simultaneously resolve complex SVs, phase variants, and evaluate methylation patterns even in complex regions of the genome. They also demonstrate the utility of LRS for evaluating X-linked conditions where phenotypic manifestations may depend on the degree of X-inactivation. Furthermore, our findings support the broader development of LRS-based methods for other conditions with limited clinical testing options.

## Supplementary Material

Supplement 1

## Data Availability

Sequence data will be available in the AnVIL under accession number phs003047.
