# Supplementary material for: Long-Read Sequencing is Required for Precision Diagnosis of Incontinentia Pigmenti
Source: Res Sq. 2025 Jan 30:rs.3.rs-5811417. Preprint. [Version 1] doi: 10.21203/rs.3.rs-5811417/v1 (PMC11838753; doi:10.21203/rs.3.rs-5811417/v1)
Supplement: Supplement 1 [file NIHPPrs5811417v1-supplement-1.pdf]

SUPPLEMENT

CASE DESCRIPTIONS

Family 1

A female neonate (V:1) was born to a 31-year-old G1P1 mother (IV:1) via Caesarean section at 40 2/7 weeks due to failure to progress after an uncomplicated pregnancy. The neonate's mother had developed a papular, erythematous rash at 2 days of age, which evolved into brown patches characteristic of incontinentia pigmenti (IP) by several months of age; she was diagnosed with IP at 10 months of age and this rash had entirely disappeared by adulthood.[1] The neonate's maternal aunt (IV:3) also presented with rash in the neonatal period and, in adulthood, continued to report thin hair and ridged nails. The neonate's maternal grandmother (III:1) had a similar rash as a neonate that evolved into brown patches and received a clinical diagnosis of IP at 1 year of age; she also has misshapen teeth and a history of five miscarriages. The neonate's maternal great-grandmother (II:1) had bald spots on the head (unknown if she had a rash), a history of seizures, and greater than three miscarriages; she also had an unaffected daughter and son. The maternal great-great-grandmother had at least five daughters (unknown if others were affected), two sons, and it is unknown if she had any miscarriages. Prior clinical genetic evaluation of the proband's mother included a negative next-generation ectodermal dysplasia sequencing panel performed on peripheral blood that included *EDA, EDAR, EDARADD*, and *IKBKG* (Fulgent Genetics, Temple City, CA).

Following an uncomplicated delivery, the neonate was discharged on day of life 3 in good health. That same day, her parents observed several self-resolving episodes of left arm and leg shaking. The next day, the neonate's pediatrician observed left upper extremity shaking with right head deviation at a routine appointment and referred her to the emergency department. In the emergency department, multiple brief episodes of insuppressible, left-sided shaking were observed and suspected to be seizures. The neonate was started on phenobarbital and prophylactic acyclovir and transferred to a tertiary care center. Upon arrival, the neonate was intubated for respiratory support in the setting of apnea. A neurology consultation and preliminary EEG revealed electroclinical seizures with onset in the left hemisphere, midline, and right hemisphere of the brain, possibly secondary to strokes. She was noted to have an erythematous papulovesicular rash **(Fig 1A)**, thought to be possibly suggestive of early IP in the context of the family history. A brain MRI on day of life 5 revealed areas of diffusion restricted in the cerebral and cerebellar hemispheres, including the thalami and left basal ganglia, that did not involve specific arterial territories. This presentation was thought to be consistent with cerebral arteriopathy secondary to IP and the neonate was started on corticosteroids, which have reportedly been successful in managing IP-related central nervous system disease.[6] The neonate's seizures subsequently became refractory, requiring four antiepileptics. Follow-up brain imaging revealed progression of several areas of injury consistent with a prognosis of moderate

to severe deficits in domains including motor function, language, and cognition. Ultimately, the family decided to transition the neonate to comfort care and she died at 13 days of age.

Family 2

A female neonate (II:1) was delivered via vaginal delivery at 39 weeks' gestation, at home with the assistance of a midwife, to a 24-year-old primigravida mother. The mother (I:1) had been treated with an 18-day course of valacyclovir for previous history of *Herpes simplex* virus (HSV) infection. The medication was stopped 2 weeks prior to delivery and the mother had no active lesions at the time of delivery. Family history was unremarkable. Apgar scores were: 4, 8, and 10 at 1, 5, and 10 minutes, respectively. Birth measurements were unremarkable. Initial exam was notable for bullous and crusted lesions on the extremities, and she was admitted to the NICU shortly after birth due to concern for HSV infection. She remained afebrile and within 18 hours of admission, the bullous lesions on extremities had transitioned into small scaly patches as well as multiple tiny hyperpigmented macules **(Fig 2A)**. Head ultrasound was normal except for a left sided tiny germinolytic cyst. Blood and cerebrospinal fluid cultures were negative for HSV, as were cultures from mucosal surfaces and lesions. She was treated with acyclovir during her admission, but it was discontinued after 4 days of therapy at time of discharge.

At a dermatology outpatient visit at 14 days of age, her exam revealed a linear, vesicular eruption with papules and streaky brown hyperpigmentation. A punch biopsy was taken that showed necrotic keratinocytes. At 2 months of age, a dilated eye exam with scleral depression of the peripheral retina was normal without signs of abnormal pigment changes. The patient's skin cleared by 7–8 weeks of age with some residual pigment on the inner thighs. She breastfed well and grew normally. Brain MRI at 15 months was normal, and the germinolytic cyst seen on head US was not apparent. At 18 months of age, she was evaluated for suspected seizures that were determined to be nonepileptic paroxysmal events. She was developmentally delayed, nonverbal and nonambulatory, and had esotropia and hyperopic astigmatism.

When reexamined in the Genetics clinic at 19 months of age, she was nondysmorphic and hypotonic. She had one small oval area of alopecia behind the right ear and no areas of skin hypoplasia. She had scattered hyperpigmentation on both arms, right abdomen and left and right thighs and right calf in linear, reticulated, and swirling patterns. Her nails and teeth were normal. She wore glasses for esotropia. She had bilateral metatarsus adductus, with a cast on her left foot. She did not have seizures and had a normal EEG. She engaged in repetitive stereotypic movements suggesting early autism and had global developmental delay. Her mother reported regression of milestones starting at 6 months of age. At 19 months she was not walking or bearing weight and she did not point or engage in imaginative play; she spoke no words but communicated with sounds and could use two signs. No other family members had a similar phenotype **(Fig 2B)**.

Family 3

Family 3 was identified during preconception evaluation of a 25-year-old Gravida 0 female with a personal and maternal family history of IP **(Fig 2E)**. IP had been clinically diagnosed over three generations, affecting the proband (III-1), her mother (II-1), maternal grandmother (I-1), and her brother (III-3), who also had Klinefelter syndrome (47,XXY). The proband's prenatal history was uncomplicated. Postnatal findings included blistering at birth, followed by a hyperkeratotic rash evolving into swirling hyperpigmented whorls along Blaschko's lines, most prominent in the lower extremities. As an adult, she had sparse eyelashes and eyebrows, nail ridges, hypodontia, and characteristic hairless skin lesions with linear, atrophic, hypopigmented areas **(Fig 2F)**. Her growth, development, and schooling were unremarkable, and she denied ocular, retinal, or seizure manifestations. Her younger brother (III-3) also showed progressive IP-related skin changes from birth. A skin biopsy and subsequent karyotype confirmed IP and revealed Klinefelter syndrome. Both the mother (II-1) and maternal grandmother (I-1) exhibited typical IP skin findings and skin appendage abnormalities, and both had multiple miscarriages.

SUPPLEMENTAL METHODS

Clinical testing

Family 1: Initial clinical testing was performed from peripheral blood-derived DNA at GeneDx (Gaithersburg, MD) and included capillary sequence analysis of *IKBKG* coding regions and long-range PCR for the recurrent exon 4–10 deletion in the neonate as well as exome sequencing on the proband's mother. Both were nondiagnostic.

Family 2: Initial clinical testing was performed from peripheral blood-derived DNA at GeneDx (Gaithersburg, MD) and included capillary sequence analysis of *IKBKG* coding regions and long-range PCR for the recurrent exon 4–10 deletion and was nondiagnostic.

Family 3: The proband (III-1) underwent molecular analysis of the *IKBKG* gene through a CLIA-certified laboratory (sema4, NY), including oligonucleotide aCGH array and next-generation sequencing supplemented by Sanger sequencing. All results were negative. X chromosome inactivation studies demonstrated extreme skewing. During preconception care, the proband pursued one round of in vitro fertilization (IVF) with PGT-SR (preimplantation genetic testing for structural rearrangements) due to her partner's known familial balanced translocation (involving chromosomes 1 and 8). A karyotype performed shortly after birth revealed the brother (III-3) to have Kleinfelter's syndrome, no additional genetic testing was performed for this individual.

DNA extractions, library preparation, and sequencing

Family 1: Long-read sequencing (LRS) was performed on the proband's mother (IV:1), maternal aunt (IV:3), and maternal grandmother (III:1) with DNA isolated from whole blood using the Monarch HMW DNA Extraction Kit (NEB #T3050L) following the manufacturers' instructions. DNA for the proband's unaffected brother (V:3) was extracted from blood by a clinical laboratory; extraction methods were not available. DNA was quantified on a Qubit using the dsDNA High Sensitivity Assay Kit (ThermoFisher); DNA quality was assessed using a NanoDrop Spectrophotometer (ThermoFisher) and Agilent Femtopulse. Two libraries for sequencing were prepared using the Oxford Nanopore (ONT) Ligation Sequencing Kit (SQK-LSK110) starting with 2.5 μg of HMW DNA in 48 μL of water with the following modifications: the DNA repair step was held at 60°C for 30 min instead of 5 min and the ligation step was done for 1 hr instead of 10 min. Libraries for sequencing were loaded onto an R9.4.1 flow cell on an Oxford Nanopore PromethION 24 running Minknow 22.03.4. For all three samples, approximately 800 ng of prepared library was loaded onto the flow cell for sequencing and allowed to run for approximately 24 hr before washing and reloading two times. A fourth library from the proband's mother was loaded onto a new R9.4.1 flow cell and run for 72 hr in

order to increase coverage. DNA from the proband (V:1) was isolated from a blood sample post-mortem. Because only 1 µg of DNA was available for sequencing, it was sheared to an average length of 20 kbp using a Megaruptor 3, then prepared for sequencing as above and loaded onto a single PromethION flow cell and allowed run for 72 hr with no reloads. Additional coverage for the *IKBKG* locus from the proband's mother was obtained using adaptive sampling on the PromethION platform. Briefly, libraries for sequencing were prepared as above with no shearing performed. Approximately 800 ng of library was loaded on a R9.4.1 PromethION flow cell and a 4-Mbp region around the *IKBKG* locus was selected for targeting (chrX:152,000,000–156,000,000, GRCh38). After 24 hr, the flow cell was washed and reloaded then run for another 24 hr.

Family 2: DNA from the proband and mother was isolated from whole blood using the Puregene DNA Purification from Blood kit (Qiagen) according to the manufacturer instructions for large volume extractions from whole blood. DNA was quantified as described above. Libraries for sequencing were prepared using the ligation sequencing kit (SQK-LSK114, ONT) following the manufacturer's instructions and approximately 950 ng of library was loaded onto R10.4.1 flow cells and allowed to run for approximately 24 hr before washing and reloading two times. After identification of the mosaic inversion in the proband we prepared a second library for sequencing from only the proband and performed adaptive sampling as described above in order to increase the number of reads spanning the inversion breakpoint.

Family 3: DNA from the proband and her brother were isolated from saliva using prepIT.L2p Oragene Saliva Extraction kit (DNAGenotek) according to manufacturer instructions. DNA was resuspended with Tris-EDTA buffer and allowed to incubate for 48 hours at 4°C or 24 hours at room temperature before quantifying. DNA was quantified as described above. Libraries for sequencing were prepared using the ligation sequencing kit (SQK-LSK114, ONT) following the manufacturer's instructions and approximately 950 ng of library was loaded onto a R10.4.1 PromethION flow cells. Adaptive sampling was run as described in Family 1. For both individuals, the flow cell was washed and reloaded after 24 hours of sequencing.

Data analysis

FAST5 or POD5 data from R9.4.1 flow cells were base called with Guppy 6.3.2 (ONT) using the super accurate model with CpG methylation (dna_r9.4.1_450bps_sup_5mC.cfg), while POD5 data from R10.4.1 flow cells were base called using Dorado 0.5.0 (ONT) using version 4.3.0 of the 5mCG_5hmCG model. Reads were aligned to GRCh38 using minimap2,[7] phased single nucleotide (SNV) and indel variants were called using Clair3 using either the R9 or R10 model[7], and LongPhase was used to generate a phased BAM file[8]. The *IKBKG* locus was then visually inspected using IGV[8] **(Fig S1)**, which allowed us to group reads by phase and visualize haplotype-specific methylation patterns **(Fig 1E, S2, S4, S5)**. For all individuals, SNV and indel

variants were annotated using VEP (version 111),[9] followed by filtering for variants with allele frequency < 0.5% based on gnomad4.[10]

Family 1: The structure of the complex SV was elucidated using reads from the proband's mother (IV:1) generated by both whole-genome LRS and adaptive sampling. SAMtools[11] was used to isolate reads > 100 kbp that mapped in the 1-Mbp region surrounding *IKBKG* (chrX:154,000,000–155,000,000, GRCh38) and were assigned to the haplotype with the *IKBKG* exon 8–10 deletion. Multiple split reads were identified that partially mapped to unique sequence outside of the segmental duplications containing either *IKBKG* or *IKBKGP1*. These split reads contained the deletion of *IKBKG* exons 8–10 as well as an inversion **(Fig S3A)**. Because the *IKBKG* exon 8–10 deletion began within an *Alu* in intron 7 (chrX:154563012-154563328) **(Fig S3B)**, we suspected that an unequal exchange event between *Alu* elements led to the formation of the complex SV **(Fig 1D)**. We isolated the sequence of the *Alu* from the mother and, using BLAT[12] determined that it mapped partially to an *Alu* element (*AluSq*) between *IKBKGP1* exons 7 and 8 (chrX:154641109-154641424) and partially to an *Alu* (*AluSq2*) proximal to *IKBKGP1* exon 10 (chrX:154638746-154639046). This suggested that the most likely first step in the formation of this complex SV was a deletion of *IKBKGP1* exons 8–10 by an unequal exchange event between these two *Alu* elements. A subsequent exchange between an *Alu* (*AluSq*) within *IKBKG* intron 7 and the novel *Alu* in *IKBKGP1* resulted in the deletion of *IKBKG* exons 8–10 and neighboring inversion. This mechanism is similar to that proposed for the formation of the common exon 4–10 deletion[1] as well as for a deletion of exons 3–10.[13]

Family 2: After alignment, visual analysis of *IKBKG* in the proband (II:1) did not identify any obvious SVs, and analysis of the annotated VCF file did not reveal any pathogenic SNV or indel variants missed by prior testing. Examination of phased reads revealed several reads that were split within intron 2 of *IKBKG*, with the split read mapping approximately 150 kbp away within an intron of *GAB3* and oriented in such a way that suggested an inversion **(Fig 2C)**. Further analysis of phased reads revealed most reads assigned to the non-maternal haplotype, which fully spanned *IKBKG*, while others were split at the presumed intron 2 inversion breakpoint, suggesting a mosaic inversion. No reads assigned to the maternal haplotype showed evidence of the inversion. After generating additional data from the proband using adaptive sampling, we recovered several reads that spanned the inversion breakpoint and the nearby *G6PD-IKBKG* CpG island, all of which were methylated and derived from the non-maternal haplotype. Methylation fractions for plotting were generated using modkit v0.2.0 (Oxford Nanopore). The CpG islands were selected for those that have characteristic X chromosome inactivation patterns as previously described **(Fig S5A)**.[14] PCR confirmation of the inversion breakpoint was performed as described below.

Family 3: The exon 5–10 deletion was identified by visual analysis of reads aligned to *IKBKG* using IGV. After phasing, all reads with the deletion in the proband were assigned to a single haplotype, while the other haplotype had no reads with a deletion. Examination of the nearby CpG island spanning *G6PD* and *IKBKG* revealed complete methylation of the same haplotype that contained the deleted reads **(Fig S5B)**. Examination of the proband's brother who was known to have Kleinfelter's syndrome (47,XXY) revealed the same deletion, with most reads carrying the deletion assigned to a single haplotype. The shorter average read lengths recovered from the brother likely resulted in some reads with the deletion being incorrectly assigned to the other haplotype. Examination of the same nearby CpG island revealed complete methylation of the haplotype carrying the deletion. The deletion was likely mediated by approximately 1 kbp homologous sequences identified as self-chain alignments, which overlap both deletion breakpoints **(Fig S6)**.[15]

Methylation analysis: To evaluate chromosome-wide methylation patterns, haplotagged BAM files for each sample were generated and modbam2bed (Oxford Nanopore) was used to estimate the percentage of methylated reads at each CpG dinucleotide. The output bed files were filtered for individual CpGs within predefined CpG islands on the X chromosome. We then calculated the mean percent methylation and the standard error of the mean for each CpG island on the X chromosome for both haplotypes. CpG islands were filtered for sites in common between family members, where the absolute difference in percent methylation between haplotypes was ≥50%. The mean percent methylation for each CpG island was plotted for each haplotype from the four confirmed 46,XX individuals from Family 1 **(Fig S4)**, the proband and mother from Family 2 **(Fig S5A)** and both confirmed individuals from Family 3 **(Fig S5B)**. All analysis was done using R version 4.1.3.

Validation of LRS findings

Family 1: Clinical long-range PCR was performed using peripheral blood samples from the proband's mother (IV:1) and maternal grandmother (III:1) as well as a male miscarriage (V:2) of the proband's mother via standard techniques using two sets of primers: 5'-CCTGCCCCATTTTATCCAGC-3' (chrX:154554888–154554907) and 5'-GTTGTGTGATGGCCCTGAAG-3' (chrX:154567599–154567618).

Family 2: The mosaic inversion identified in the proband was evaluated using PCR. Primers were designed using Primer3[16] and 5 reactions were run: two laboratory control samples, the mother (I:1), the affected proband (II:1), and water as a negative control. LongAmp Taq 2X Master mix kit (New England Biolabs) was used with amplification performed in a 50-μL reaction consisting of 2 μL of forward primer (IKBKG_L2: 5'-CAGTGAAGTTTGCCTGGGAG-3') at 10 μM, 2μL of reverse primer (IKBKG_R2: 5'-GTTTGGTCTTTGTTTCATGGTCA-3') at 10 μM, 25 μL of LongAmp Taq 2X Master mix, 19

μL of nuclease-free water, and 2 μL of DNA. Reactions were amplified on a SimpliAmp Thermal Cycler (Applied Biosystems). The machine was programmed for initial denaturation at 94°C for 30 seconds, followed by 30 cycles of 30 seconds at 9°C for denaturation, 45 seconds at 55°C for annealing, and 30 seconds at 65°C for extension, and a final extension for 10 minutes at 6 °C. 17 μL of each PCR product was combined with 5 μL of 1X E-Gel sample loading buffer, and 20 μL of each was loaded into a well of an Invitrogen 1% precast agarose gel, with the E-Gel 1 Kb Plus DNA Ladder in the far left lane. This was then run on an E-Gel Power Snap Electrophoresis machine for 10 minutes.

Family 3: PCR validation of the deletion was not performed.

SUPPLEMENTAL FIGURES

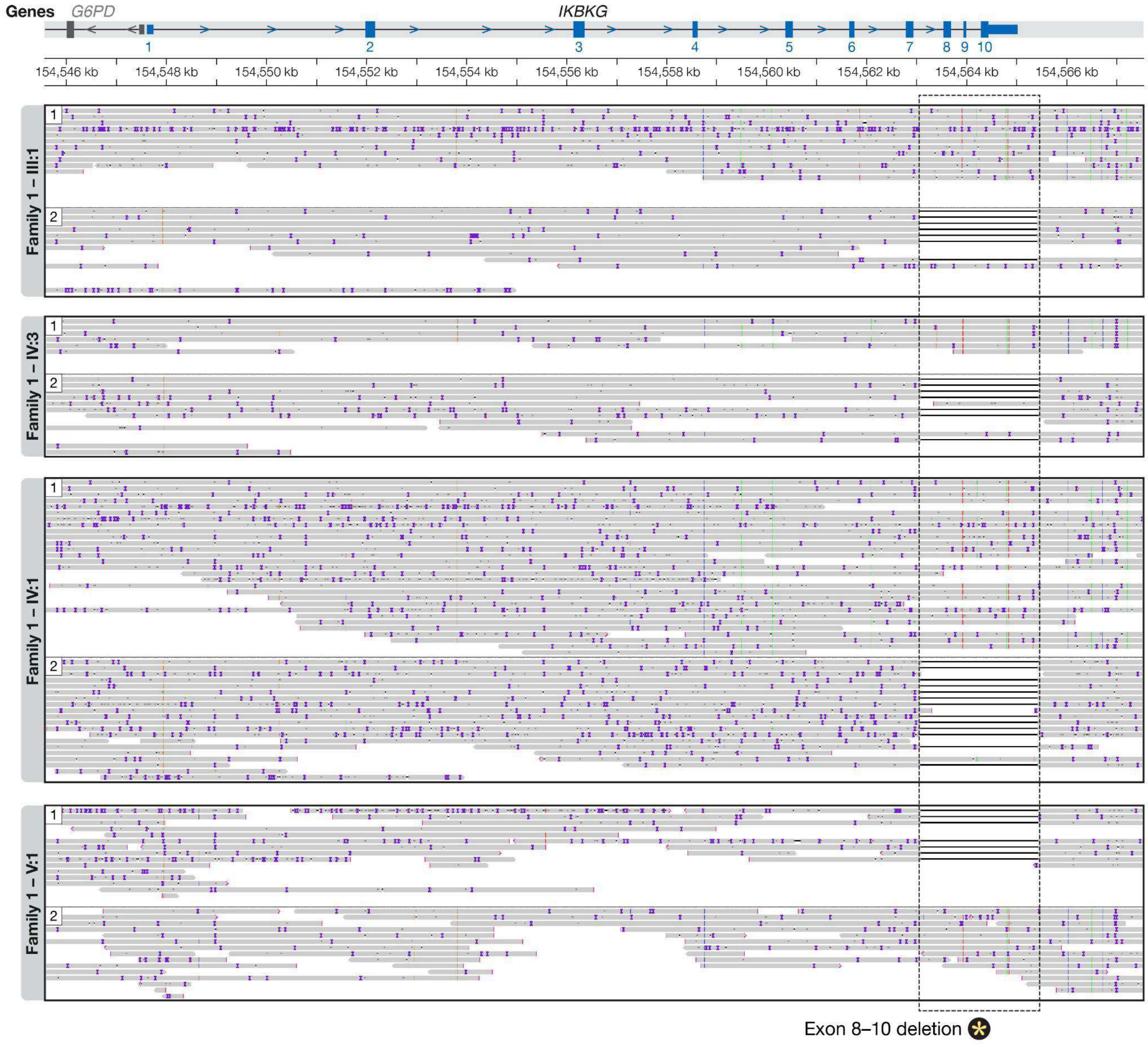

Figure S1. All four affected females in Family 1 carry a deletion of *IKBKG* exons 8–10. IGV view of aligned LRS data grouped by phase shows that all four affected females **(Fig 1B)** share a ~2,350-bp deletion (dashed box) on one haplotype that includes exons 8–10 of *IKBKG*.

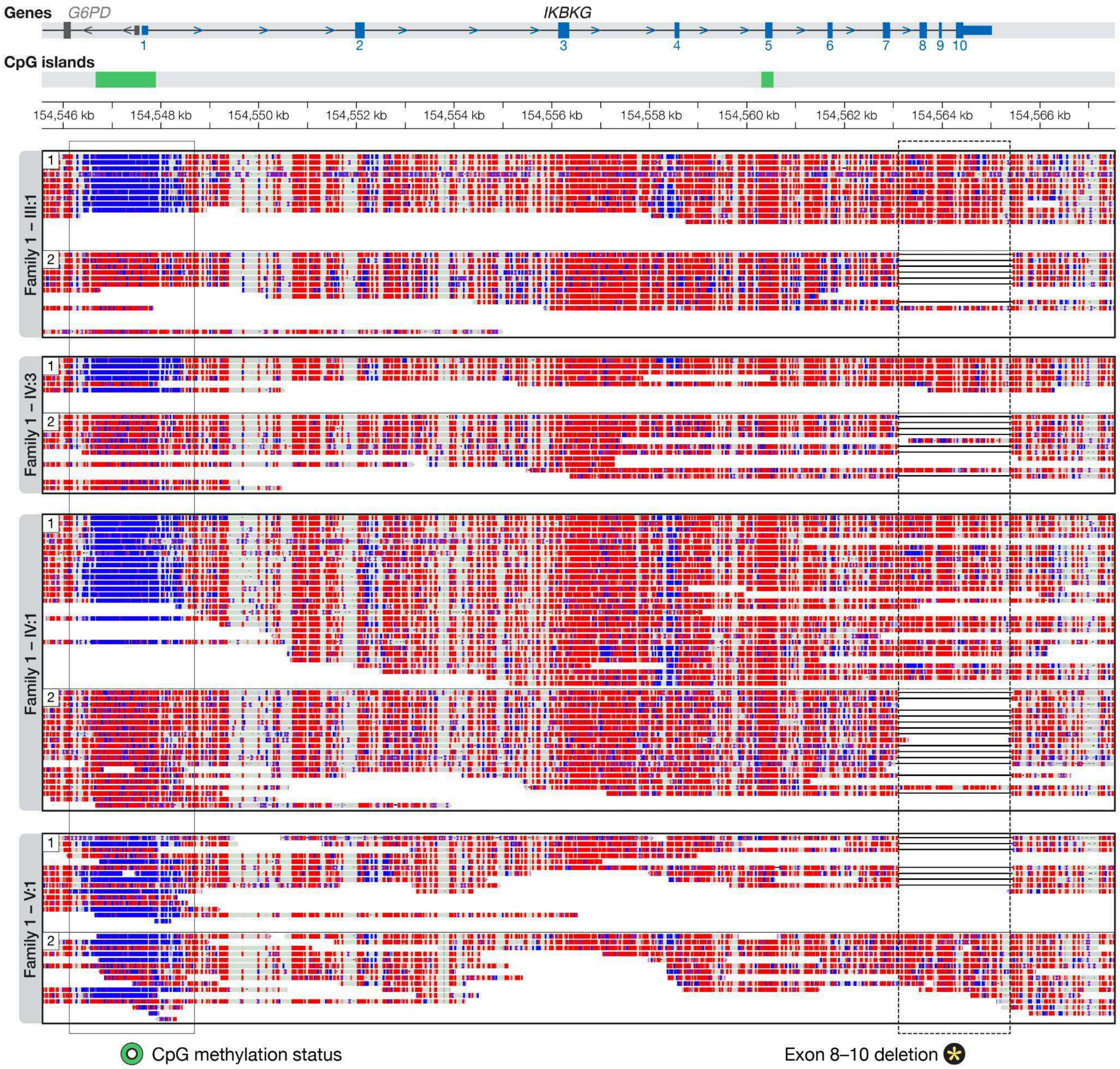

Figure S2. CpG methylation patterns from Family 1 show skewed X-inactivation in mildly affected females. IGV view of aligned LRS data grouped by phase as shown in Fig S1, with bases colored by CpG methylation status. A methylated (inactive) CpG is shown as red, unmethylated (active) CpGs are blue. The CpG island shared by *G6PD* and *IKBKG* is outlined by the gray box. The three mildly affected family members **(Fig 1B)** have completely inactivated the haplotype carrying the *IKBKG* exon 8–10 deletion (dashed box) while the severely affected proband (V:1) has mixed inactivation of both haplotypes.

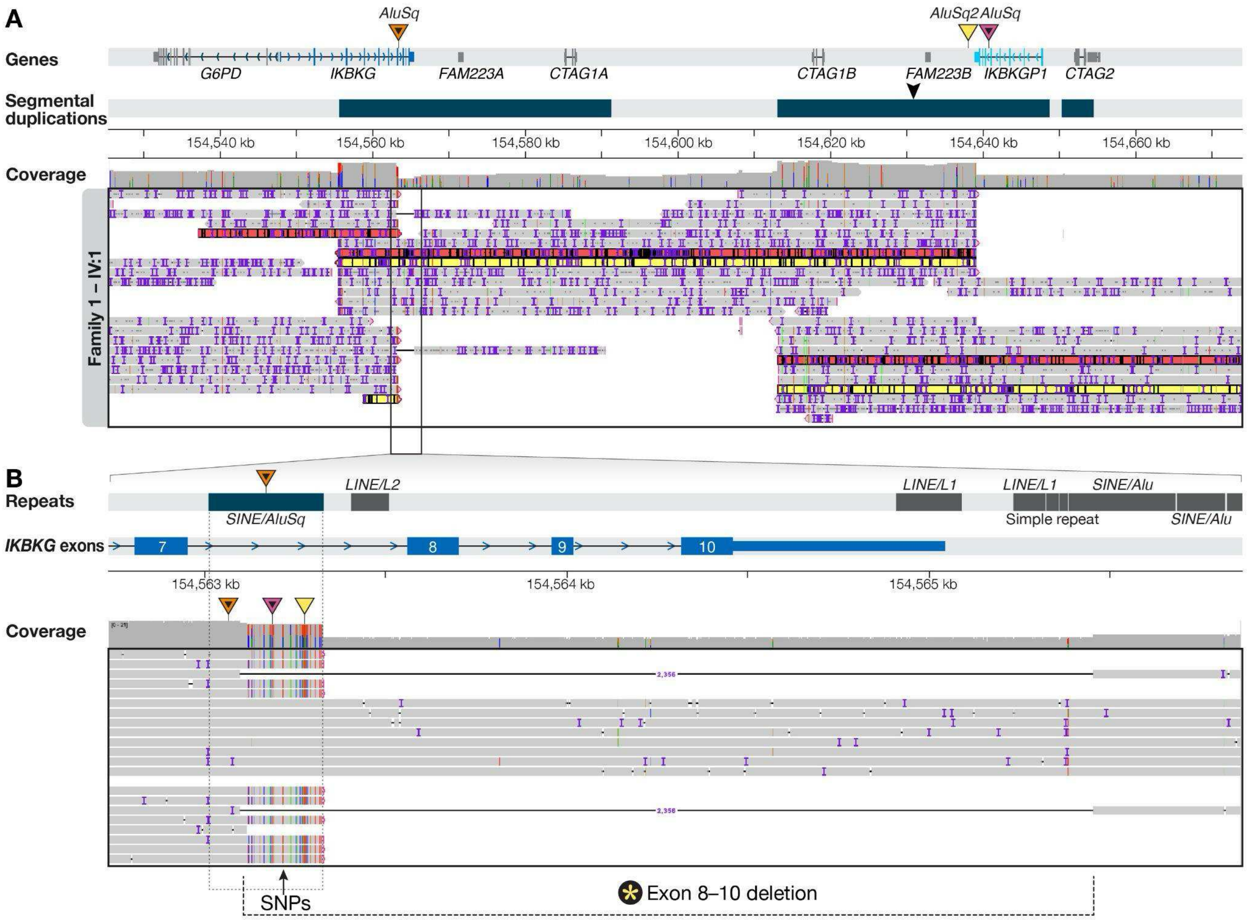

Figure S3. An inversion in Family 1 is associated with the *IKBKG* exon 8–10 deletion.
(**A**) Reads from the proband's mother (IV:1) generated by both whole-genome LRS and adaptive sampling are shown here. Reads longer than 100 kbp that mapped to the haplotype with the *IKBKG* exon 8–10 deletion were isolated and supported the presence of an inversion associated with the *IKBKG* exons 8–10 deletion. For example, the reads highlighted in red (179,909 base-pairs) and yellow (135,397 base-pairs) begin mapping either within *G6PD* or *IKBKG*, are then split and inverted, then split again and properly map beyond the large distal segmental duplication (arrowhead). Analysis of the read depth (coverage) incorrectly suggests the presence of two duplications associated with the inversion in the regions covering *IKBKG*, *CTAG1B*, and FAM223B; this is an artifact caused by sequence from split reads being mapped multiple times. (**B**) Analysis of DNA sequences that mapped to the haplotype carrying the *IKBKG* exon 8–10 deletion revealed a high number of SNPs in an *Alu* element (*AluSq*) at the proximal deletion breakpoint. BLAT of the sequence of that *Alu* partially mapped to two other *Alu* elements in the *IKBKGP1* pseudogene, an *AluSq* within intron 7 of the pseudogene and an *AluSq2* just proximal of exon 10 of the pseudogene (Figure 1D).

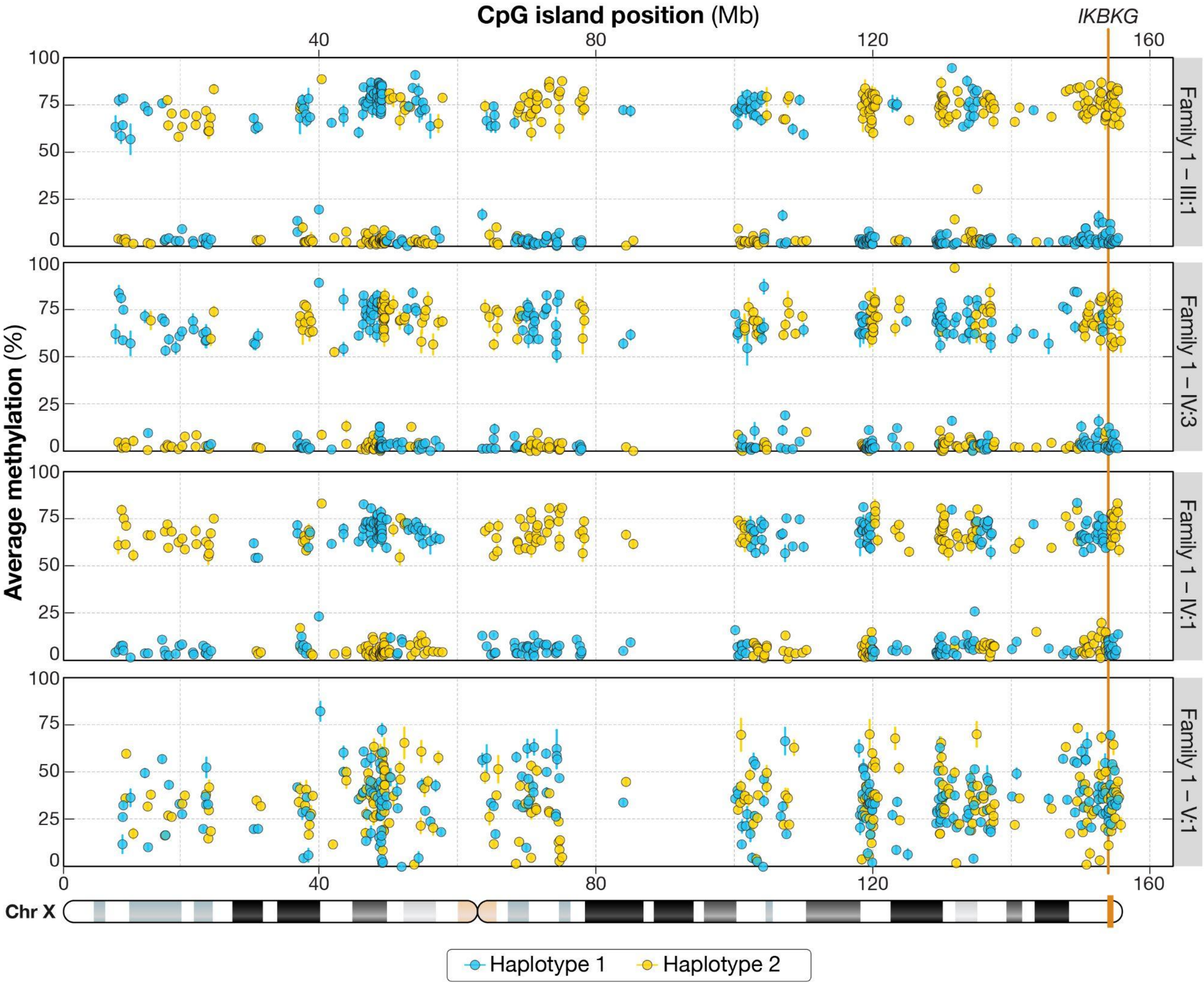

Figure S4. X-chromosome methylation patterns demonstrate skewed X-inactivation in mildly affected females from Family 1. Haplotype-resolved CpG island methylation patterns show that the three mildly affected females have skewed X-inactivation while the severely affected proband has random inactivation across the entire chromosome. Fewer data points are available in the proband due to overall lower coverage. Haplotype-specific methylation percentage does not reach 100 in completely inactivated haplotypes because not all CpG sites within a CpG island are called as either methylated or unmethylated. Haplotype numbers switch based on phase block assignment and do not represent a contiguous assembly. Error bars represent one standard error in each direction.

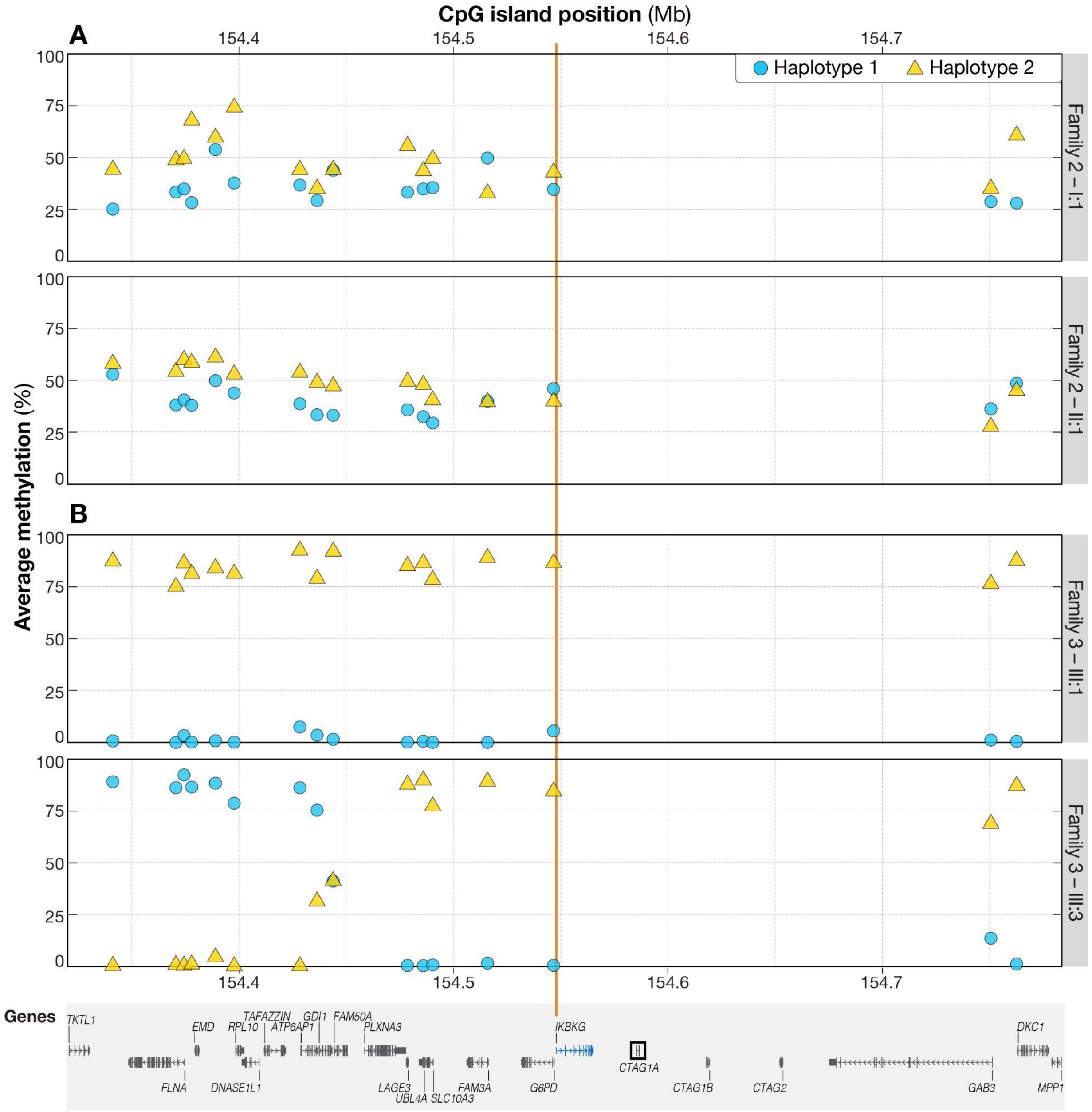

Figure S5. X chromosome methylation patterns for individuals from Families 2 and 3. The region shown is chrX:154320129–154783633. **(A)** In Family 2, in which the proband (II:1) carries a mosaic inversion bisecting *IKBKG*, X chromosome methylation does not appear skewed in either the mother (I:1) or proband. **(B)** Both the proband (III:1), and a mildly affected male with Kleinfelter's (III:3) have skewed X-inactivation for the region shown.

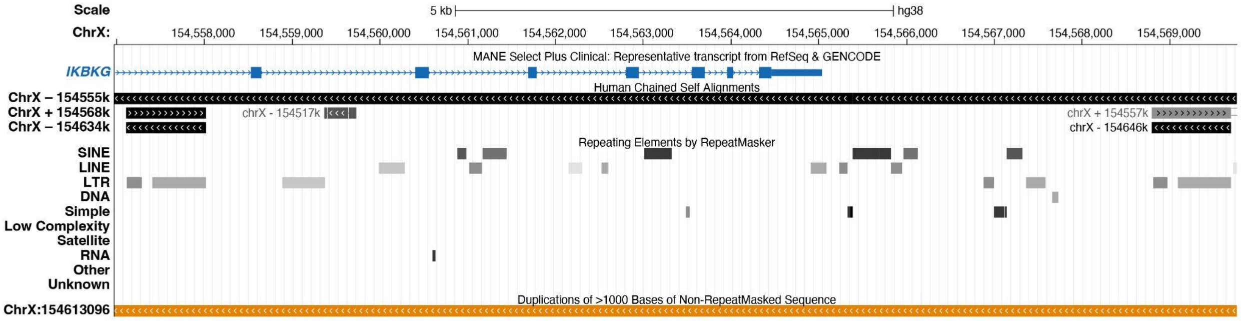

Figure S6. The 11.7-kbp deletion in Family 3 likely occurred by unequal exchange between two similar LTR elements. The UCSC view shows the self-chained alignment track, which demonstrates high homology between the two LTR elements annotated in the RepeatMasker track.

SUPPLEMENTAL REFERENCES

1. Aradhya, S. *et al.* A recurrent deletion in the ubiquitously expressed NEMO (IKK-γ) gene accounts for the vast majority of incontinentia pigmenti mutations. *Hum. Mol. Genet.* **10**, 2171–2179 (2001).
2. Frans, G. et al. Conventional and Single-Molecule Targeted Sequencing Method for Specific Variant Detection in IKBKG while Bypassing the IKBKGP1 Pseudogene. *J. Mol. Diagn.* **20**, 195–202 (2018).
3. Pipko, N. et al. Genome sequencing reveals novel IKBKG structural variants associated with incontinentia pigmenti. *Br. J. Dermatol.* ljae462 (2024) doi:10.1093/bjd/ljae462.
4. Logsdon, G. A., Vollger, M. R. & Eichler, E. E. Long-read human genome sequencing and its applications. *Nat Rev Genet* 1–18 (2020) doi:10.1038/s41576-020-0236-x.
5. Zhou, W. et al. Increased genome instability in human DNA segments with self-chains: homology-induced structural variations via replicative mechanisms. Hum. Mol. Genet. **22**, 2642–2651 (2013).
6. Ogasawara, K. et al. Corticosteroid Therapy in Neonatal Incontinentia Pigmenti With Asymptomatic Cerebral Lesions. *Pediatr. Neurol.* **99**, 85–87 (2019).
7. Li, H. Minimap2: pairwise alignment for nucleotide sequences. *Bioinformatics* **3**, 321 (2018).
8. Thorvaldsdóttir, H., Robinson, J. T. & Mesirov, J. P. Integrative Genomics Viewer (IGV): high-performance genomics data visualization and exploration. *Brief Bioinform* **14**, 178–192 (2013).
9. McLaren, W. et al. The Ensembl Variant Effect Predictor. *Genome Biol* **17**, 122 (2016).
10. Karczewski, K. J. et al. The mutational constraint spectrum quantified from variation in 141,456 humans. *Nature* **581**, 434–443 (2020).
11. Li, H. et al. The Sequence Alignment/Map format and SAMtools. Bioinformatics **25**, 2078–2079 (2009).
12. Kent, W. J. BLAT—The BLAST-Like Alignment Tool. *Genome Res* **12**, 656–664 (2002).
13. Fusco, F., D'Urso, M., Miano, M. G. & Ursini, M. V. The LCR at the IKBKG Locus Is Prone to Recombine. *Am. J. Hum. Genet.* **86**, 650–652 (2010).
14. Gustafson, J. A. et al. High-coverage nanopore sequencing of samples from the 1000 Genomes Project to build a comprehensive catalog of human genetic variation. *Genome Res.* **34**, gr.279273.124 (2024).
15. Kent, W. J., Baertsch, R., Hinrichs, A., Miller, W. & Haussler, D. Evolution's cauldron: Duplication, deletion, and rearrangement in the mouse and human genomes. *Proc. Natl. Acad. Sci.* **100**, 11484–11489 (2003).
16. Untergasser, A. et al. Primer3—new capabilities and interfaces. *Nucleic Acids Res.* **40**, e115–e115 (2012).
